# Profiles of Alcohol Use Disorders, Problematic Pornography Use, and Compulsive Sexual Behavior: The Role of Mental Health, Moral Disapproval, Thought Suppression, and Self-Concealment Among Israeli–Arab Adolescents

**DOI:** 10.1007/s10508-025-03179-2

**Published:** 2025-06-23

**Authors:** Misk Kabaha, Yaniv Efrati

**Affiliations:** Faculty of Education, Bar-Ilan University, Ramat Gan, 5290002 Israel

**Keywords:** Alcohol use disorder, Compulsive sexual behavior, Problematic pornography use, Network analysis, Latent profile analysis, Adolescents

## Abstract

Alcohol use disorders (AUDs), compulsive sexual behavior (CSB), and problematic pornography use (PPU) are prevalent disorders among adolescents. Research indicates an increase in the number of adolescents engaging in daily alcohol, porn consumption, and sexual behavior, as well as an increase in the number of adolescents diagnosed with these disorders. The aim of the current study was to detect unique factors of these addictive behaviors with mental health, moral disapproval, thought suppression, and self-concealment and examine whether these factors could explain the different profile severities of AUDs, PPU, and CSB among Israeli–Arab adolescents. The sample comprised 622 Israeli–Arab adolescents (64% boys, 36% girls; ages 14–18). First, by using network analysis we found three factors: (1) alcohol use, (2) CSB, problematic pornography use, and mental health, and (3) moral disapproval, thought suppression, and self-concealment of pornography and sexual behavior. Second, by using latent profile analysis we identified five distinct profiles based on the three detected factors: (1) low alcohol use amidst high CSB and moral disapproval, thought suppression, and self-concealment; (2) low CSB, pornography, and alcohol use; (3) average CSB, pornography, and alcohol use; (4) high CSB, pornography, and alcohol use; and (5) high CSB, pornography, and alcohol use amidst low disapproval, thought suppression, and self-concealment levels. The findings underscore that unique profiles strongly correlate with different addictive behaviors among adolescents. The current research contributes to the field by offering a more personalized approach to comprehending addictive behaviors and culture-related risk factors among Israeli–Arab adolescents.

## Introduction

During adolescence, individuals often engage in various behaviors, including those that are risky (Bozzini et al., 2020; Ciranka & van den Bos, 2021). Such activities are common among adolescence and include alcohol consumption (Jones et al., 2020), pornography exposure, and engaging in sexual activity (Landripet et al., 2025), which may start as exploratory but can escalate to substance use disorders or behavioral addictions (Maggs et al., 2023; Van Rooij et al., 2014).

The rise of digital technology and the internet have significantly increased access to sexually explicit material and alcohol, normalizing their use among adolescents. This trend is underscored by studies documenting early exposure to pornography (Peter & Valkenburg, 2016) and alcohol (Smit et al., 2018). While there is considerable research on these issues in adults (Wright, et al., 2024) and young adult (Jepsen et al., 2024), a significant gap remains in understanding how these exposures affect adolescents with cultural sensitivity, particularly their potential to disrupt social or occupational functioning and cause distress (Brand et al., 2022; Zou et al., 2017). In fact, to date, limited research has been conducted among Israeli–Arab adolescents with regard to alcohol (Baron-Epel et al., 2015), pornography, and sexual behavior (Jabareen et al., 2023), particularly concerning the onset of addictive behaviors. In the current study, we adopted a different and novel perspective. We first estimated a network analysis between all main study measures: frequency of use; severity of alcohol use disorders (AUDs), problematic pornography use (PPU), and compulsive sexual behavior (CSB); and thought suppression, self-concealment, and moral disapproval of alcohol consumption, pornography, and sexual behavior). We then examined the factors we detected and performed a latent profile analysis (LPA). Finally, we compared the detected profiles with regard to the following background measures: age, socioeconomic status (SES), gender, and religiosity. By doing so, the current study could enable the detection of adolescents at risk of developing AUD, PPU, and CSB.

### Alcohol Use Disorders

Alcohol consumption represents a significant global public health risk, with rates varying globally from 14 to 83% in the past 30 days (Vashishtha et al., 2021). As young individuals make their way through adolescence, and into their 20s and beyond, the likelihood of alcohol use, akin to other drug usage, escalates (Patton et al., 2018). The National Survey on Drug Use and Health indicates that, by the age of 12, approximately 1 in 100 (1%) adolescents have consumed alcohol in the preceding month (White, 2020). This prevalence increases to nearly 1 in 4 (23%) by the age of 17 (CBHSQ, 2019). In a recent study by Farnia et al. (2024) based on the Global School-based Student Health Survey, the overall prevalence of adolescent alcohol consumption was reported at 25.2%, with rates of 28.3% in boys and 22.4% in girls. The highest prevalence was observed in Seychelles (57.9%), while the lowest was in Tajikistan (0.7%), highlighting the impact of religious and legal restrictions on alcohol consumption. For instance, in Islamic countries like Tajikistan and Iran, where alcohol is prohibited and considered sinful, the prevalence was notably lower at 5.7% over the past year and 1% over the past week (Amin‐Esmaeili et al., 2018). This underscores the influence of religious beliefs and legal penalties on alcohol use. In Israel, Arab adolescents face a unique situation; while legally permitted to consume alcohol due to Western influences, religious prohibitions within the Muslim community discourage its use. Among Arab adolescents, the percentage of students who reported getting drunk in the 10th grade was 5.8%, and in the 11th–12th grades, it increased to 14.9%. Additionally, they indicated consuming five servings of alcoholic beverages within a few hours at least once in the last 30 days; in the 10th grade, the rate of such behavior was 9.3%, and in the 11th–12th grades, the rate was 9.2% (Harel-fish, 2023). Certain adolescents may develop AUDs, reporting impaired control over their alcohol consumption. They may chronically exhibit a heavy and often escalating pattern of alcohol use despite serious detrimental costs to their overall health, and to the lives of their family members and friends. AUDs are defined by a loss of control over alcohol consumption, compulsive use, and experiencing negative emotional states during periods of non-use, often following a chronic and relapsing course. Diagnostically, both DSM and ICD require multiple criteria to be met within a 12 month period due to alcohol's adverse psychological, biological, behavioral, and social impacts (Carvalho et al., 2019). In 2016, AUDs affected about 5.1% of the global population, with a higher prevalence in men (8.6%) than in women (1.7%), particularly in high-income and upper-middle-income countries (Rehm et al., 2019; World Health Organization, 2018). Despite the significant public health consequences, various AUDs remain among the most undertreated mental disorders (Carvalho et al., 2019). Specifically, AUDs are particularly prevalent, with conditional probabilities of first onset peaking at approximately 15 years of age, and a median age of onset at 19 (McGrath et al., 2023).

### Problematic Pornography Use and Compulsive Sexual Behavior

Human beings are sexual beings, capable of sexual responses from childhood (O’Sullivan & Thompson, 2014). Adolescence, however, signals the commencement of substantial changes in sexual and reproductive maturity, coinciding with significant transformations in cognitive, emotional, and social functioning (Backes & Bonnie, 2019). Throughout this developmental phase, the most prevalent sexual activities include the consumption of pornography and engagement in sexual behaviors (Ballester-Arnal et al., 2023). In Israel, among Arab adolescents, approximately 44% reported sexual behaviors such as the use of pornography, intimate physical contact, and sexual intercourse (Jabareen et al., 2023). Adolescents may develop PPU or CSB due to impaired control over their consumption or behaviors, as these tendencies correlate with developmental changes associated with adolescence (Efrati, 2020; Hegde et al., 2022). This stage of life includes identity exploration and increased impulsivity, making adolescents particularly vulnerable to addictive behaviors (Castellanos-Ryan et al., 2025). Factors like insecure attachment, lack of emotion regulation (Efrati, 2023b), and easy access to digital media and online sexual behavior further heighten these risks (Efrati & Amichai-Hamburger, 2021).

PPU is increasingly recognized as a common manifestation of CSB, characterized by uncontrollable consumption patterns that lead to significant distress and functional impairment (Kraus et al., 2018). Studies indicate that about 10% of adolescents are at risk for PPU, correlating with frequent pornography use and elevated sexual arousal (Bőthe et al., 2021). CSB, characterized by the World Health Organization as an impulse control disorder (WHO, 2019), affects an estimated 3–10% of adults and 12–18% of adolescents, highlighting a prevalent global mental health concern (Briken et al., 2022; Grubbs et al., 2023). The condition includes a range of behaviors from pornography use to excessive sexual fantasies, often resulting in substantial distress or dysfunction. There is ongoing debate whether CSB should be classified as an impulse control, compulsivity-related, or an addictive disorder. Under the behavioral addiction framework, CSB is seen as involving repetitive behaviors that initially provide gratification but soon lead to severe consequences due to tolerance and withdrawal issues (Gola et al., 2022; Potenza et al., 2017). Despite extensive documentation, research on PPU and CSB among Israeli–Arab adolescents remains limited, indicating a significant gap in the current literature.

Islam strictly prohibits nonmarital sexual activities, emphasizing this in several Quranic verses (Ali, 2016). For instance, Quran 17:32 warns against fornication as a grievous wrong, instructing Muslims to avoid paths that could lead to such acts. Similarly, Quran 23:5–6 restricts sexual relations to marriage, reinforcing the sanctity of marital bonds. Moreover, Quran 24:2 prescribes severe penalties for unmarried individuals who engage in sexual activities, underscoring the serious consequences of such actions within the Islamic faith. Despite these clear teachings, research on Muslims' perceptions of pornography remains limited, highlighting a significant gap in studies focused on how religiosity influences pornography consumption among Muslims (Grubbs et al., 2017). This gap suggests a need for more culturally tailored approaches in counseling and research to better address and understand the nuances of PPU and CSB within Muslim communities.

These phenomena—namely, AUDs, PPU, and CSB—are influenced by distinctive social and normative factors. A significant number of Israeli–Arab adolescents identify themselves with religion or traditional Muslim values as a crucial aspect of their lives (Dwairy, 2019). In Islam, behaviors such as the consumption of alcohol, the viewing of pornography, and engagement in sexual activity (before marriage) are strictly prohibited (Michalak et al., 2006; Gesser-Edelsburg & Abed Elhadi Arabia, 2018). Consequently, by avoiding initiation into these behaviors, many practicing Israeli–Arabs are shielded from AUDs, PPU, and CSB. However, for Israeli–Arabs, the initiation of alcohol use, pornography viewing, or sexual behavior may lead to heightened vulnerability to AUDs, PPU, and CSB due to the taboo nature of these behaviors in their community. This societal disapproval can intensify feelings of guilt and conflict, increasing the risk of these disorders. Influenced by traditional Islamic gender roles, attitudes towards pre-marital sexual activity are distinctly shaped; boys experience fewer social constraints compared to girls who are taught to value virginity highly (Abboud et al., 2015; Jabareen & Zlotnick, 2023; Muhammad et al., 2017). Research from Arab nations reflects a higher incidence of pornography use among males, a trend observed across conservative cultures where premarital sex is generally forbidden (Eljawad et al., 2021). This could be attributed to religious and cultural prohibitions that drive secretive sexual explorations (Uğur et al., 2024). Similarly, boys are noted to consume more alcohol than girls, although the overall usage remains comparatively low due to religious prohibitions (Baron-Epel et al., 2015).

Affected Israeli–Arabs commonly face challenges in openly discussing their struggles with AUDs, PPU, and CSB within their communities due to fears of rejection or ostracization (Hassan et al., 2021). Additionally, collective denial and prevalent beliefs that the problematic use of alcohol, pornography, and sex does not exist within Israeli–Arab communities contribute to under-recognizing the true extent of addictive behaviors in this minority population. This lack of acknowledgment can result in spiritual crises and a poorer prognosis for AUDs, PPU, and CSB. Therefore, we hypothesized that culture-related risk factors among Israeli–Arab adolescents (thought suppression, moral disapproval, and self-concealment) would be associated with addictive behaviors and decreases in mental health.

### Thought Suppression

The conscious inhibition of thoughts or reasoning represents a self-monitoring strategy characterized by efforts to manage psychological stress through distancing oneself from unwanted thoughts (Wenzlaff & Wegner, 2000). Thought suppression, defined as the attempt to ignore undesirable thoughts, is one of the most common approaches for handling objectionable thoughts, particularly when sharing them openly is not considered an option (Brockman et al., 2017; Gross & John, 2003). Efforts to suppress specific thoughts often lead to a rebound effect, where the suppressed thought becomes more prevalent (Abramowitz et al., 2001; Rassin et al., 2000; Wenzlaff & Luxton, 2003). Research in Iran on individuals with AUDs and nicotine dependence revealed that negative affect and thought suppression indirectly influence cravings and dependencies via desire thinking (Khosravani et al., 2022). Furthermore, studies on PPU highlight the ineffectiveness of thought suppression as a strategy among members of a "rebooting" forum (Fernandez et al., 2021). Efrati and colleagues suggest that suppressing sexual thoughts not only influences the development of CSB (Efrati & Spada, 2024) but also mediates between general metacognition and CSB (Efrati et al., 2020, 2021), exacerbating the issue in contexts with strong religious prohibitions (Efrati, 2019).

### Moral Disapproval

Individuals navigate their social worlds by adhering to moral norms, as outlined in moral foundations theory, which delineates core values such as care, fairness, loyalty, authority, and purity (Graham et al., 2018). These values, often rooted in religious or cultural beliefs, are viewed as sacred by some, making them non-negotiable regardless of the situation (Kivikangas et al., 2021). For instance, someone who values fairness highly would never accept a bribe to commit an injustice, reflecting the stability of their moral and religious convictions (Haidt, 2012). This consistency in moral values is crucial for understanding behaviors such as alcohol use, pornography consumption and sexual behavior, particularly within specific demographic groups. Research, such as that by Cho & Yang (2023), indicates that feelings of guilt associated with violating these moral norms can deter behaviors like AUDs in some people by encouraging less harmful drinking habits or delaying the initiation of drinking. However, in those with established AUDs, guilt may exacerbate problems rather than prevent them, illustrating how moral disapproval's effectiveness can vary widely (Grynberg et al., 2017).

The moral incongruence model of pornography use, developed by Grubbs and Perry (2019), provides an integrative explanation for varying responses to pornography use. This model illustrates that individuals might experience issues with pornography stemming from either dysregulated use, which aligns with CSB diagnostic guidelines, or from conflicts between their pornography use and their moral or sexual values, known as pornography problems due to moral incongruence (PPMI). It is important to note that moral incongruence highlights the disparity between personal beliefs about the moral disapproval of pornography use and actual consumption behaviors (Grubbs and Perry, 2019; Huțul & Karner-Huțuleac, 2023; Lewczuk et al., 2021; Ostrander, 2021). Distress arising solely from moral disapproval should not lead to a CSB diagnosis unless accompanied by dysregulated use. In the current study, Israeli–Arab adolescents exhibit moral incongruence, highlighting a clash between their personal beliefs, religious prohibitions, and cultural norms against behaviors such as pornography use, sexual activity, and alcohol consumption, and their actual practices in these areas. Therefore, it's crucial to understand AUDs, PPU, and CSB within their specific cultural and moral contexts, as these significantly shape individuals' interpretations and responses to such behaviors.

### Self-Concealment

In this study, the cultural aspect of self-concealment, defined as the deliberate withholding of personal information deemed negative or distressing (Larson & Chastain, 1990), was explored. This behavior often correlates with the suppression of troubling or potentially embarrassing details about oneself (Fridlander et al., 2012). Self-concealment is posited to strain self-regulatory capacities by tapping into limited cognitive resources necessary for impulse control and decision-making (Baumeister et al., 2000), thereby potentially exacerbating behavioral addictions (Magsamen-Conrad & Greene, 2014). While self-concealment can serve to protect one's self-image and prevent conflict (Bok, 2011), it's also associated with increased depression, anxiety, and social withdrawal (Larson & Chastain, 1990; Yukawa et al., 2007). In line with Lazarus's coping framework, self-concealment is an avoidant emotion-focused coping strategy, leading individuals to rely more on internal coping mechanisms (Sefi et al., 2021), which may limit effective problem-solving and increase reliance on maladaptive behaviors (Uysal, 2020) like addictive behaviors (Chen et al., 2023).

A recent study by Baudat et al. (2022) found that adolescents who often conceal information from their parents tend to exhibit higher rates of problematic alcohol use, suggesting a connection between secrecy and alcohol-related problems. In Arab–Israeli communities, where alcohol consumption is both prohibited and stigmatized, adolescents must hide their drinking habits, which can strain their mental well-being and potentially lead to AUDs. Similarly, in the book "The Psychology of Secrets," Kelly (2002) discussed how adolescents often confide in trusted friends about their pornography use while avoiding disclosure to their parents, highlighting the significant effort involved in maintaining such secrecy. Among Israeli–Arab adolescents, prohibitions on viewing pornography or engaging in sexual behaviors outside of marriage necessitate secrecy. This concealment, similar to hiding alcohol use, may lead to PPU or CSB, diminishing mental well-being.

### The Current Study

The main goal of this study was to identify distinct profiles of culture-related risk factors associated with the severity of AUDs, PPU, and CSB in Israeli–Arab adolescents. To do so, a data-driven person-centered approach was employed, in which latent profiles of individuals based on the following were examined: prevalence of drinking alcohol, watching pornography, engaging in sexual behavior, AUDs, PPU, CSB, moral disapproval, thought suppression, self-concealment, mental health, gender, and religiosity. The aim of the study was to use network analysis to explore the interplay between addictive behaviors and culture-related risk factors, so as to investigate whether these factors could predict the severity of addictive behavior profiles. Specifically, the hypothesis was that Israeli–Arab adolescents with high-risk culture-related factors would exhibit greater severity of AUDs, PPU, CSB, and negative mental health.

## Method

### Participants and Procedure

The study’s sample consisted of 622 Israeli–Arab adolescents from the general community of Israel’s Triangle area (a concentration of Israeli–Arab towns and villages adjacent to the Green Line, located in the eastern Sharon plain among the Samarian foothills), with 399 boys (64%), 222 girls (36%), and one who did not identify themselves (0.2%). The participants were between the ages of 14–18 years (*M* = 16.68, *SD* = 1.12). The rationale for the selected age range was that it represents a period of extensive transformations (Crone et al., 2012) and the onset of various psychopathologies, including addictive behaviors (Holmbeck et al., 2006; McGrath et al., 2023). In terms of SES, 2.4% of the participants described their status as “very bad,” 14% as “bad,” 74% as “good,” and 9.5% as “very good.” Regarding religion, 145 participants (23%) self-reported as religious, 457 participants (73%) as traditional, and 20 participants (3.2%) as secular.

Adolescents aged 14–18, regardless of their religious affiliation from the Triangle area in Israel, were eligible to participate in this study. The primary objective was to explore alcohol use, pornography consumption, and sexual behavior among Israeli–Arab adolescents. Recruitment efforts included posting notices on bulletin boards and engaging with online forums. Parents, once they had given their consent, forwarded the study link to their children. The sample excluded adolescents who were receiving clinical care and/or who had a diagnosis of persistent mental health conditions. Participants were informed that the study focused on addictive behaviors among Israeli–Arab adolescents. Parents provided consent for participants under 18, whereas those aged 18 provided their own consent. To ensure confidentiality, participants completed measures in a quiet home environment. The measures, presented randomly with specific instructions, were administered through the online survey platform Qualtrics. Of the 803 adolescents contacted, 622 (77.5% response rate) completed the questionnaire, taking an average of 17 min. All measures were in Arabic, the native language of most Israeli–Arabs. It should be noted that the response rate led to a non-identical ratio between boys and girls. Upon survey completion, participants were electronically debriefed on the study's objectives and were thanked for their participation.

### Measures

The translation of the assessment tools into Arabic was performed using a back-translation method, as advocated by recognized translation guidelines (Beaton et al., 2000; Hambleton & Zenisky, 2010; Maneesriwongul & Dixon, 2004). Initially, two specialized psychologist teams translated each instrument into Arabic, producing two separate versions. These were then reviewed and merged into a single version per instrument, which was subsequently back-translated into English by a professional to ensure fidelity to the original. Any discrepancies found were discussed and rectified, ensuring the translated scales retained the intended meanings of the originals.

#### Alcohol, Pornography Use, and Sexual Behavior Frequency

Alcohol use, pornography use, and sexual behavior frequency were assessed separately, with one item addressing each behavior, using an 11-point scale: “Do you drink alcohol?” Do you watch pornography?” “Do you behave sexually (have sex, masturbate, wear revealing clothing, etc.)?” The scale ranged from 0 (*not at all*) to 11 (*more than 7 times a week*). Initially, the items were translated into Arabic by a speaker proficient in both languages. They were then back-translated by the first author.

#### Alcohol Use Disorder Identification Test (AUDIT-10; Saunders et al., 1993)

The 10-item version of the AUDIT covers three primary domains of alcohol use: alcohol consumption (Items 1 to 3, measuring frequency, typical quantity, and heavy episodic drinking/HED, respectively; e.g., Item 2:” How many drinks containing alcohol do you have on a typical day when you are drinking?”); alcohol dependence (items 4 to 6, measuring impaired control, failure to meet expectations, and morning drinking, respectively; e.g., Item 4:” How often during the last year have you found that you were not able to stop drinking once you had started?”); and negative consequences (Items 7 to 10, measuring guilt feelings, blackouts, injuries, and others’ concerns, respectively; e.g., Item 4: “Have you or someone else been injured because of your drinking?”). Each item was scored between 0 and 4 points, with five response options for items 1 to 8 and three response options for items 9 and 10. The total score on the AUDIT (with a range of 0–40 points) and distribution by the AUDIT resulted in four risk categories. In this study, 227 (36%) participants fell into the category of “low-risk drinking”; 64 (10%) participants fell into the category of “risk of hazardous use”; 44 (7.1%) participants fell into the category of “risk of harmful alcohol use”; and 287 (46%) participants fell into the category of “risk of alcohol dependence.” The Cronbach’s alpha for this study was 0.98.

#### Problematic Pornography Consumption Scale (PPCS6-A; Bőthe et al., 2021)

The PPCS6-A assesses PPU with six items regarding the past 6 months (e.g., “When I promise myself not to watch porn anymore, I can only stop for a short time”). Each item represents one component of Griffiths’ (2005) six-component model of addiction (i.e., salience, tolerance, mood modification, relapse, withdrawal, and conflict). Adolescents indicated their answers on a 7-point scale ranging from (*never*) to 7 (*all the time*); higher scores indicated higher levels of PPU. The Cronbach’s alpha for this study was 0.98.

#### Compulsive Sexual Behavior Disorder Scale (CSBD7; Bőthe et al., 2023)

The CSBD-7 is a scale that comprehensively assesses compulsive sexual urges, thoughts, and behaviors and their consequences in the past 6 months along five factors corresponding to the ICD-11 diagnostic guidelines (World Health Organization, 2022).

Participants were asked to rate, on a 5-point Likert scale from 1 (*totally disagree*) to 5 (*totally agree*) the degree to which each statement characterized their feelings (e.g., negative consequences: “My sexual urges and impulses changed me in a negative way”). The Cronbach’s alpha in the current study was 0.96. The Arabic translation is available at Bőthe et al. (2023).

#### Alcohol, Porn, and Sexual Thought Suppression (Based on Wegner et al., 1987)

This variable was assessed using the Suppression Scale (Efrati, 2019) based on the White Bear Inventory (Wegner et al., 1987) and the expressive suppression scale of the Emotion Regulation Questionnaire for Children and Adolescents (ERQ–CA), developed by Gullone and Taffe (2012). Thought suppression regarding alcohol, pornography use, and sexual behavior was assessed separately with twelve items. Participants were asked to rate, on a 5-point Likert scale from 1 (*not at all*) to 5 (*very much*) the degree to which each statement characterized their feelings (e.g., Item 1: “Very often I find myself trying to suppress my alcohol/porn/sexual thoughts”; Item 11: “Sometimes I try to get involved with work or studies just to avoid all sorts of alcohol/porn/sexual thoughts”). The Cronbach’s alphas in the current study were 0.96 for thought suppression regarding alcohol; 0.94 for thought suppression regarding porn; and 0.95 for thought suppression regarding sex.

#### Alcohol, Porn, and Sexual Moral Disapproval (Grubbs et al., 2018)

To assess levels of moral or ethical objections to alcohol, porn, and sexual thoughts, we used a scale developed by Grubbs et al. (2018). The scale consists of four statements, and respondents rated their level of agreement with each statement using a 7-point Likert scale. The scale was used separately for “drinking alcohol,” “viewing pornography,” and “having sexual thoughts.” For example, “I believe that drinking alcohol/viewing pornography/having sexual thoughts is morally wrong.” The Cronbach’s alpha in the current study was 0.96 for drinking alcohol; 0.94 for viewing pornography; and 0.95 for having sexual thoughts.

#### Alcohol, Porn, and Sexual Self-Concealment (SCS; Larson & Chastain, 1990)

The Self-Concealment Scale is a self-report inventory that assesses an individual's inclination to hide personal information deemed distressing. The scale was used separately to assess self-concealment regarding alcohol, porn, and sexual behavior (e.g., “I drink alcohol/ watch pornography/engage in sexual activities, and I hide it from my parents”). The SCS includes 10 items and uses a 5-point Likert scale ranging from 1 (*strongly disagree*) to 5 (*strongly agree*) for each item. The total score is obtained by summing the responses to all items, with higher scores indicating a greater degree of self-concealment. In the present study, the measure exhibited high internal consistency with the following Cronbach’s alphas: 0.98 for alcohol; 0.98 for porn; and 0.98 for sexual behavior.

#### Mental Health Index (MHI-5; Ware et al., 1993)

The MHI-5, a subscale of the RAND SF- 36 Quality of Life Scale (Ware et al., 1993), is a nonspecific measure of mental health. The measure was used to assess well-being and the occurrence and degree of psychological distress (usually of anxiety and depression-related distress). The validity and reliability of the MHI-5 has been extensively studied in the past (e.g., Rumpf et al., 2001). In the current study, we calculated scores of mental health by averaging the relevant items. The Cronbach’s alpha in the current study was 0.85.

#### Sociodemographic Variables

Adolescents reported their age, biological sex (male, female), religiosity (secular, traditional, religious), and SES (“very good,” “good,” “bad,” and “very bad” in accordance with Israeli classifications).

### Data Analysis

The analyses were conducted on 622 Israeli–Arab participants. Before the primary analyses, we examined the normal distribution of all main study measures using a series Anderson–Darling normality tests. We also assessed the presence of multivariate outliers using the Minimum Covariance Determinant approach (performed with the *Routliers* R package). We found that all measures significantly deviated from normality (all *p* < 2.20^–16^ or lower) and that 298 observations were multivariate outliers. Accordingly, we used non-parametric statistics in our primary analyses. In addition, 2.84% of the data were missing (with 43 different patterns of missing data). We identified the type of missing data using Jamshidian and Jalal’s non-parametric Missing Completely At Random (MCAR) test. We found that the data were Missing At Random (MAR), *T*_*median*_ = 3.57, *p*_*median*_ = 0.013. Accordingly, missing data were handled with Multiple Imputations using the *mice* and *micemd* R packages.

We first estimated a network between all main study measures—frequency of use, severity of abuse (i.e., alcohol use disorder, problematic pornography use, and compulsive sexual behavior), thought suppression, concealment, and moral disapproval of alcohol consumption, pornography, and sexual behavior. We also included in the network the two clusters of mental health—well-being and psychological distress. We specifically employed the Extended Bayesian Information Criterion with Graphical Lasso estimates of polychoric (two ordinal measures), polyserial (mix of ordinal and continuous measures), and Pearson (two continuous measures) correlation matrix. The estimation process was performed with the *estimateNetwork* function of the *bootnet* R package. The network allowed the deciphering of the factorial construct (using the *EGAnet* R package) and the centrality of the variables within the network. Centrality refers to a series of measures to evaluate the function of each node (i.e., variable) within the network. We focused on the following scores: (1) Closeness—a centrality measures how fast information can spread from a given node to other reachable nodes in the network. It is calculated as the inverse of the sum of distances from a node to all other nodes. (2) Betweenness—quantifies the number of times a node acts as a bridge along the shortest path between two other nodes. It can highlight nodes significantly influencing the network, even if they are not directly connected to many other nodes. (3) Strength—In the context of network analysis, strength centrality is the sum of the weights of a node’s edges, often used in weighted networks to assess the influence or importance of a node. And (4) Expected Influence—a measure used in weighted, directed networks that calculates the expected influence of a node by summing the weights of outgoing edges and subtracting the weights of incoming edges. It provides a nuanced view of a node’s centrality, considering the direction of relationships. The network stability was appraised using bootstrapped estimations of the centrality measures (i.e., to what extent the centrality indices of the variables remain constant in different subsamples), the factorial construct (i.e., to what extent the number of factors changed in different subsamples), and variable stability (i.e., to what extent variables switched factors in different subsamples). The former analysis was conducted with the *bootnet* R package and the latter two with the *EGAnet* R package.

Following the network analyses, we used the factors we detected and performed a latent profile analysis using *tidyLPA* R package, which is based on MPlus 8.8 structural equation modeling (SEM) integration. We examined 1 to 6 possible profiles. The optimal number of profiles was determined by Akogul and Erisoglu’s (2017) Analytic Hierarchy Process (AHP), bootstrapped likelihood ratio test (BLRT), sample size of each profile, and theoretical plausibility. The AHP uses the following information criteria in its decision tree: Akaike’s Information Criterion (AIC), Approximate Weight of Evidence (AWE), Bayesian Information Criterion (BIC), Classification Likelihood Criterion (CLC), and Kullback Information Criterion (KIC). The goal of the analysis was to model the different profiles of alcohol, pornography, and sexual use and abuse among Israeli Arab adolescents.

In the final part of the analyses, we compared the profiles we detected in the following background measures: age, socio-economic status, gender (male, female), and religiosity (secular/traditional, religious). The continuous measures of age and socioeconomic status were analyzed with Welch’s analysis of variance, Holm-Bonferroni post-hoc comparisons, and partial omega-squared as the effect size. The categorical measures of gender and religiosity were analyzed with chi-square analyses of independence, post-hoc pairwise tests of independence for nominal data with false discovery rate adjustment (using the *pairwiseNominalIndependence* function of the *rcompanion* R package), and Cramer’s V as the effect size.

## Results

### Network Analysis

**Fig. 1:**
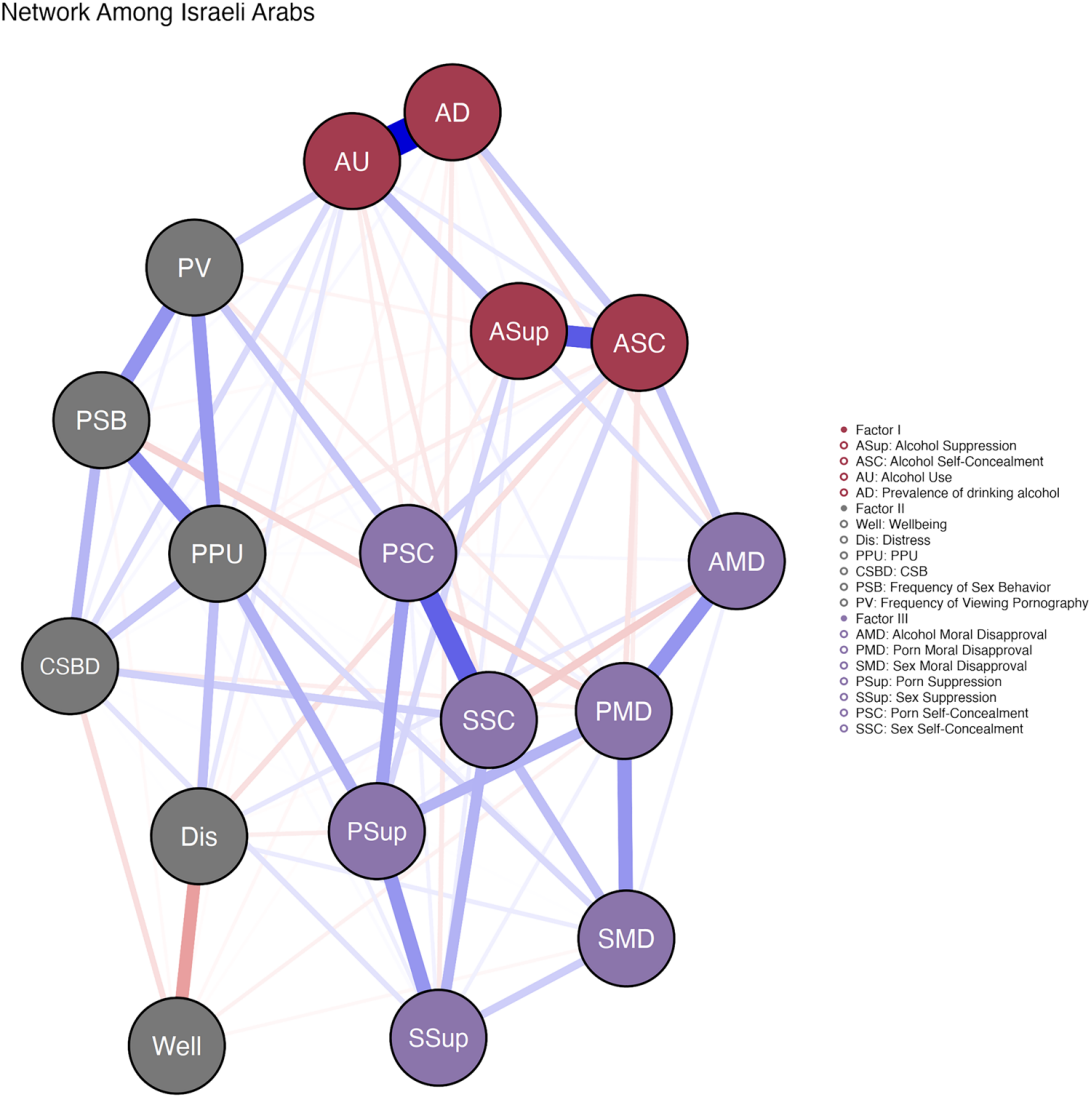
Network analysis among Israeli–Arab participants. Blue edges (i.e., paths) reflect positive associations, whereas red edges reflect negative associations. The edges’ brightness, distance, and width reflect different relative strength indicators. Nodes’ colors highlight the detected factors: alcohol use (red), CSB and mental health (gray), and moral disapproval, suppression, and self-concealment of pornography and sexual behavior (purple) (Color figure online)

**Fig. 2:**
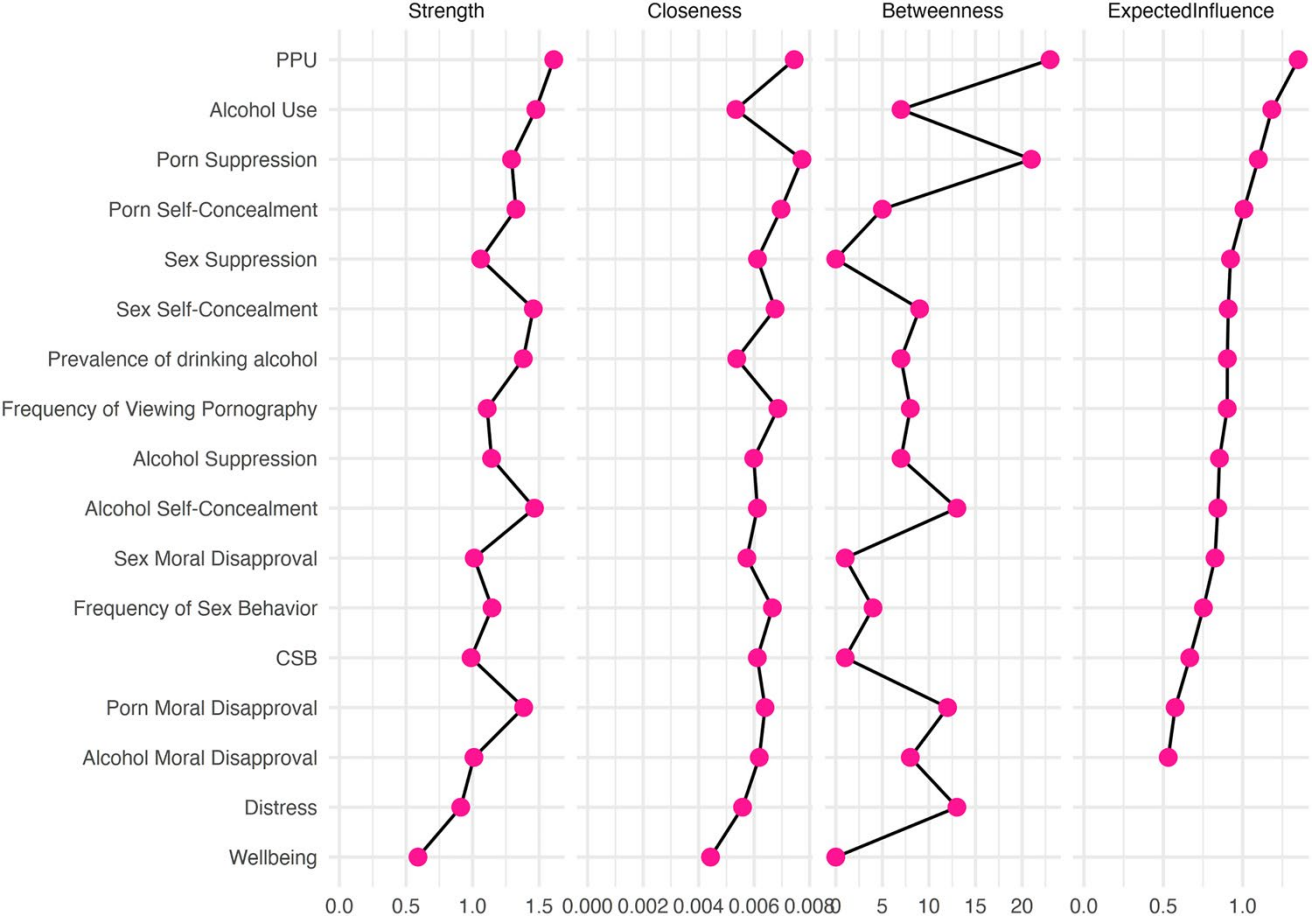
Measures of centrality. The variables in the network are sorted according to their expected influence on the network

**Fig. 3:**
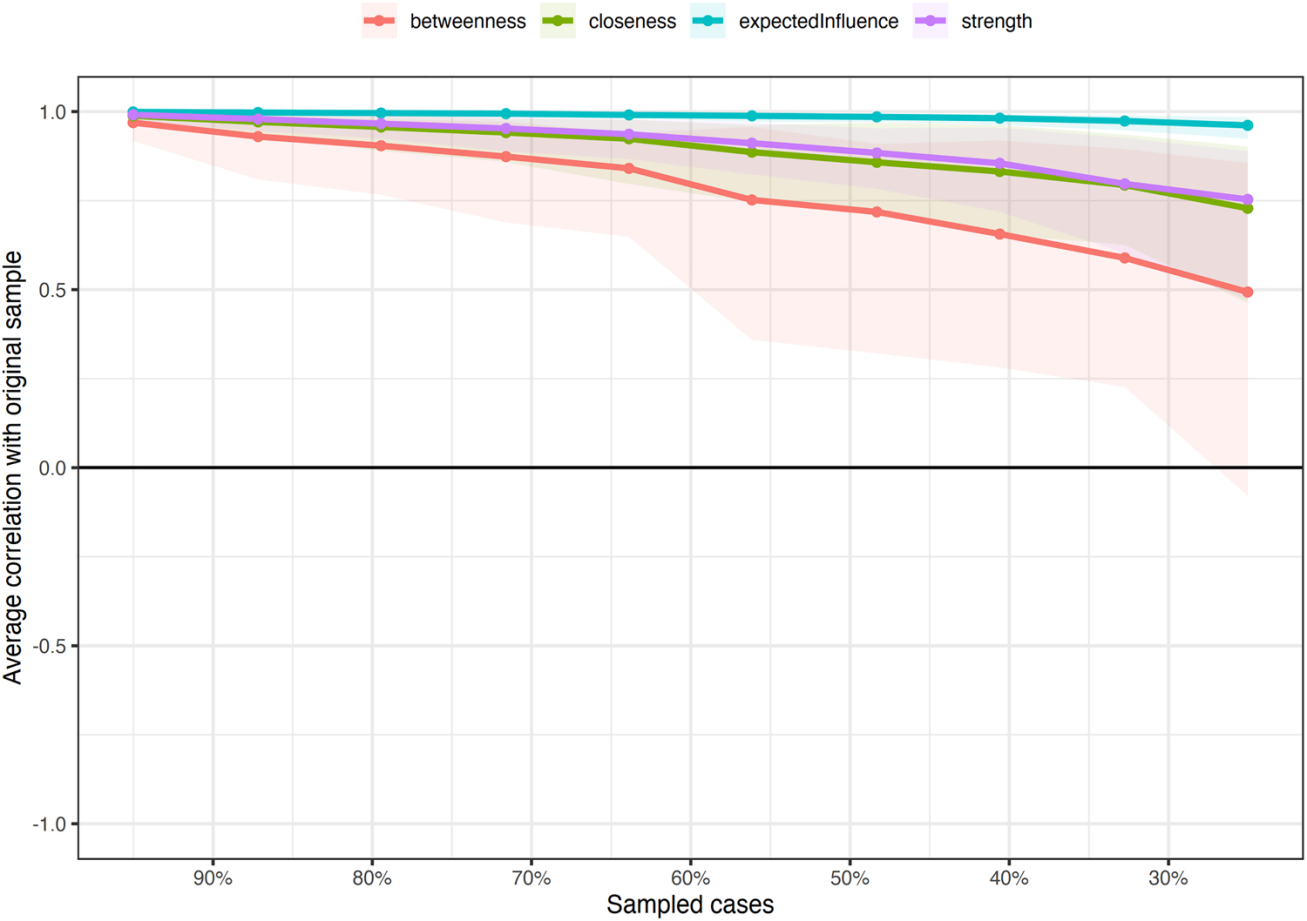
Centrality stability analyses. The stability of the Betweenness Index is below the recommended threshold of 75%

The results of the network analysis are presented in Fig. 1, the centrality scores in Fig. 2, and the centrality stability in Fig. 3. The analysis detected three factors: (1) alcohol use, (2) CSB, PPU, and mental health, and (3) moral disapproval, suppression, and self-concealment of pornography and sexual behavior. The “alcohol use” factor comprised the measures of an AUDs, the prevalence of drinking alcohol, and alcohol suppression and self-concealment. The “CSB, PPU, and mental health” factor consisted of the measures of CSB, PPU, the prevalence of viewing pornography and sexual behavior, and the measures of well-being and psychological distress. The final factor of “moral disapproval, thought suppression, and self-concealment of pornography and sexual behavior” comprised all measures of moral disapproval and the measures of thought suppression and self-concealment of pornography and sexual behavior. The bootstrapped analyses indicated that the network, factorial construct, and variables showed relatively high stability. Specifically, three out of four centrality scores had stability above 75% (with betweenness below this recommended level), 74.72% of the analyses produced a 3-factor solution (95% confidence interval between 2.1 and 3.9), and all variables had stability over 75% (i.e., tended not to switch factors in different subsamples).

The network analysis also revealed that the most influential variable within the sub-network of “alcohol use” was alcohol use disorder, of the sub-network of “CSB, PPU, and mental health” was PPU, and of the sub-network of “moral disapproval, thought suppression, and self-concealment of pornography and sexual behavior” was pornography suppression. Of note, PPU was also the most influential variable in the entire network. In addition, PPU and pornography suppression were the two variables with the highest betweenness and closeness indices. It signifies that PPU and pornography suppression act more frequently as a bridge along paths between two other nodes as well as acting as the hubs with the fastest spreading of information within the network of Israeli–Arab adolescents. In comparison, AUDs was low on betweenness and closeness, signifying that the sub-network of “alcohol use” was only lightly related to the other parts of the network—that of pornography and sexual behavior. Finally, the indicative variables of mental health are neither alcohol use nor moral disapproval, thought suppression, and self-concealment of pornography and sexual behavior. Instead, these indices are within the sub-network of “CSB, PPU, and mental health” and especially PPU and CSB.

### Latent Profile Analysis

**Fig. 4:**
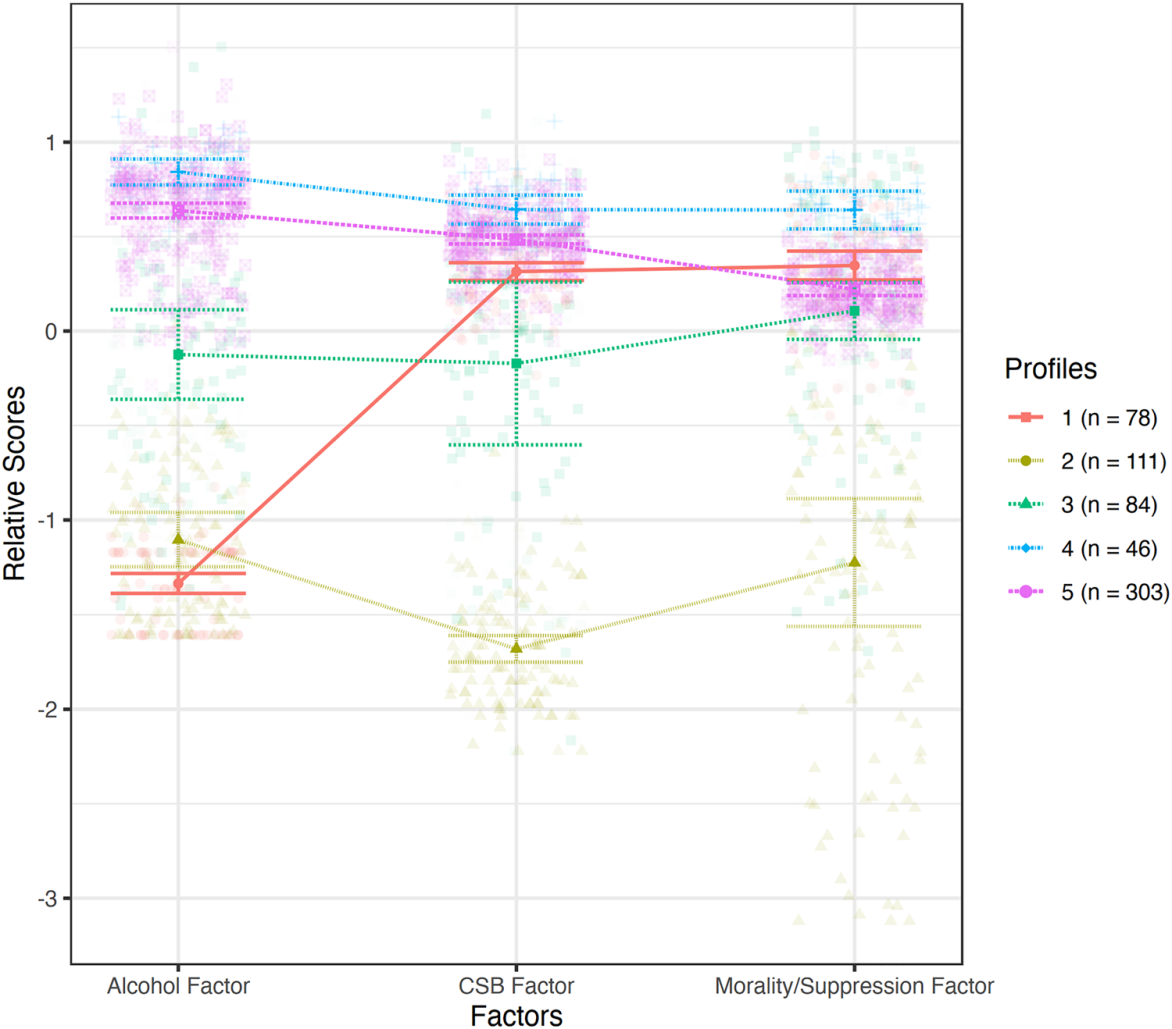
Results of the latent profile analysis. Five profiles were detected: (1) low alcohol use amidst high CSB and moral disapproval, thought suppression, and self-concealment (orange, *n* = 78, 12.5%); (2) low CSB, pornography, and alcohol use (yellow, *n* = 111, 17.8%); (3) average CSB, pornography, and alcohol use (green, *n* = 84, 13.5%); (4) high CSB, pornography, and alcohol use (blue, *n* = 46, 7.4%); and (5) high CSB, pornography and alcohol use amidst low disapproval, thought suppression, and self-concealment levels (purple, *n* = 303, 48.7%) (Color figure online)

The latent profile analysis was based on the three detected factors: “alcohol use,” “CSB, PPU, and mental health,” and “moral disapproval, thought suppression, and self-concealment of pornography and sexual behavior.” The analysis revealed that the ideal solution was 5-profiles. The Analytic Hierarchy Process (AHP) indicated that it had the lowest fit scores (with lower being better): AIC = 1989.41, AWE = 2459.03, BIC = 2140.13, CLC = 1923.23, and KIC = 2026.41. It also had a significant bootstrapped likelihood ratio test (BLRT = 155.03, *p* < 0.001), adequate sample sizes of the detected profiles (minimum sample size of 7.4%), a high entropy (90.9), and profiles with adequate theoretical differences. The five profiles were: (1) low alcohol use while high CSB and moral disapproval, thought suppression, and concealment (orange, *n* = 78, 12.5%); (2) low CSB, pornography, and alcohol use (yellow, *n* = 111, 17.8%); (3) average CSB, pornography, and alcohol use (green, *n* = 84, 13.5%); (4) high CSB, pornography, and alcohol use (blue, *n* = 46, 7.4%); and (5) high CSB, pornography and alcohol use while holding low disapproval, thought suppression, and concealment levels (purple, *n* = 303, 48.7%). The profiles are presented in Fig. 4. This profile solution highlights several implications: the most prevalent profile comprises Israeli Arab adolescents with high levels of CSB, pornography, and alcohol use while not contemplating the moral disapproval of the acts, without the need to self-conceal or suppress the sexual sides of the behavior (the need to suppress and self-conceal the use of alcohol was high). Two other less prevalent profiles comprise youth with either below-average or average levels of CSB, pornography, and alcohol use (a total of 31.3%). These profiles relate to adolescents who normally engage in these behaviors, with no signs of a disorder. The least common profile consists of Israel-Arab adolescents with high levels of CSB, pornography, and alcohol use and a high prevalence of contemplating the moral disapproval of the acts and the need to self-conceal or suppress the behaviors. Only 7 out of 100 youth show this profile. Finally, another low-frequency profile relates to adolescents who engage in CSB and pornography, thinking about the need to self-conceal or suppress the sexual sides of the behavior but shun away alcohol.

### Differences Between Profiles in Background Measures

**Fig. 5:**
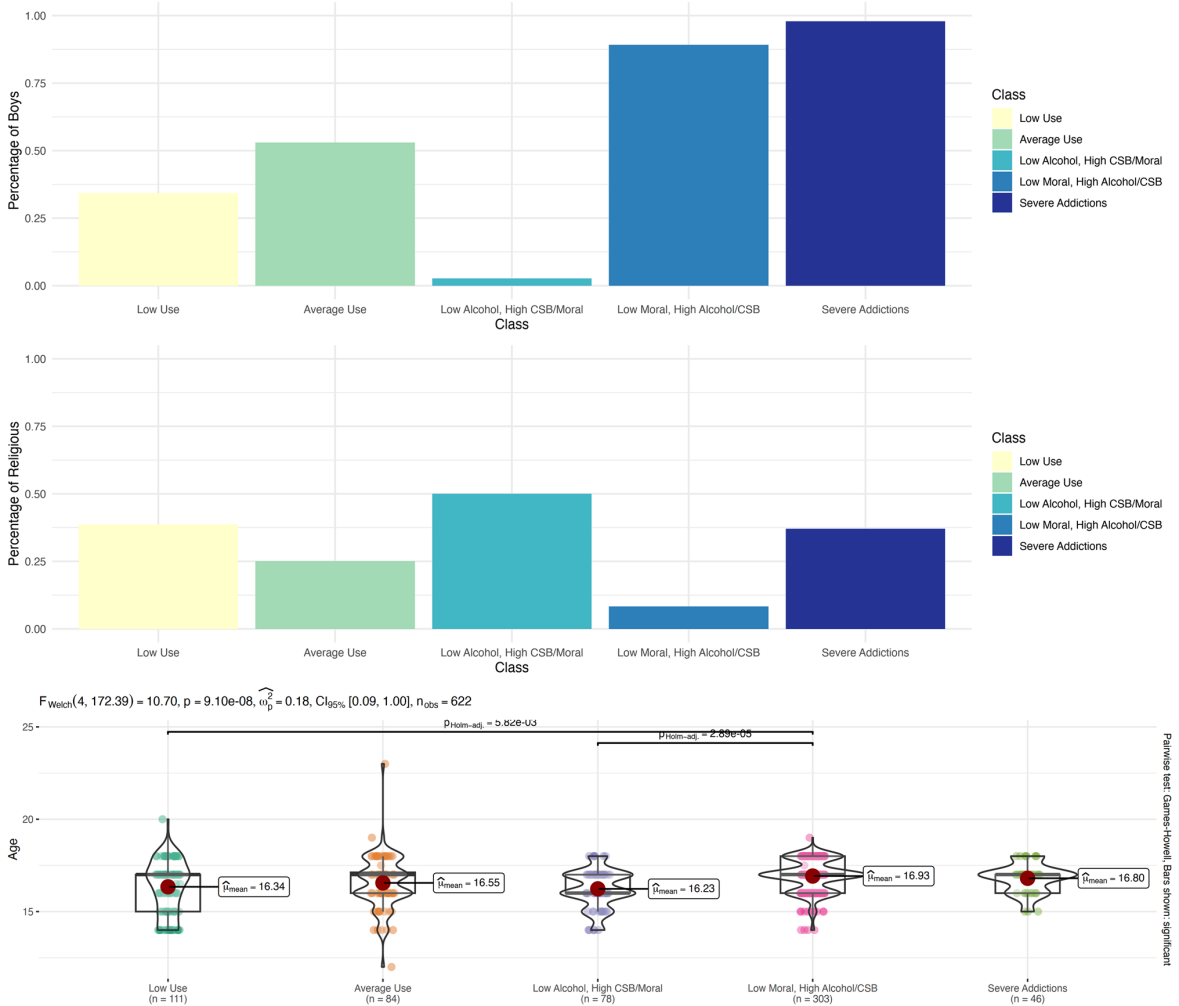
Differences in gender, religiosity, and age between the five detected profiles

The analyses indicated that the five profiles were significantly different in gender (*χ*^2^(4) = 281.42, *p* < 1.10^–59^, Cramer’s V = 0.67), religiosity (*χ*^2^(4) = 89.22, *p* < 1.92^–18^, Cramer’s V = 0.38), and age (*F*(4, 172.39) = 10.70, *p* = 9.10^–8^, *ω*^2^ = 0.18). No differences were found in SES (*F*(4, 169.38) = 0.98, *p* = 0.42, *ω*^2^ = 0.00). The pattern of differences is presented in Fig. 5. Post-hoc analysis revealed the following patterns: With respect to gender, the “average use” group had almost the same ratio of boys and girls (53% boys). In comparison, the “low alcohol, high CSB” group comprised almost entirely of girls (2.6% boys), whereas the “low use” group also had a majority of girls (34.2% boys). Conversely, the “high severity” and “high severity without contemplating on morality and suppression” groups had a majority of boys (97.8% and 89.1%, respectively). The gender ratio of all groups differed significantly at *p* < 0.01.

With respect to religiosity, the analysis indicated that the “high severity without contemplating on morality and suppression” group had the least religious adolescents (8.3%), whereas the “low alcohol, high CSB” group had the most religious adolescents (50.0%). The three other groups had an intermediate frequency of religious adolescents with no differences between the three groups in that frequency.

Finally, with respect to age, the “low use” and “low alcohol, high CSB” groups were significantly younger than the “high severity without contemplating on morality and suppression” group. Other differences were not significant.

## Discussion

The purpose of the present study was to determine whether there are different profiles with regard to the severity of the most common behaviors during adolescence, including AUDs, PPU, and CSB, among Israeli–Arab adolescents with various culture-related risks (thought suppression, self-concealment, and moral disapproval) and mental health. Previous research on addictive behaviors in adolescents has largely focused on understanding the underlying mechanisms of such behaviors (Bőthe et al., 2021) or has used clinical perspectives (Vidal et al., 2023). Conversely, only a few studies have explored profiles of AUDs (Villarosa-Hurlocker et al., 2020), PPU (Jiang et al., 2022; Zarate et al., 2023) and CSB (Efrati & Gola, 2018), and we found no studies on culture-related profiles among adolescents that could predict addictive behaviors. To address this research gap and achieve the study’s objectives, network analysis and LPA were utilized.

Via network analysis, we found three factors reflecting alcohol use and sexual behavior among Israeli–Arab adolescents. The first factor was “alcohol use,” comprising the measures of AUDs, the prevalence of drinking alcohol, thought suppression regarding alcohol, and self-concealment. Alcohol consumption among Israeli–Arabs is a unique phenomenon that sets off an internal conflict among adolescents, caught between the desire to drink and the prohibition of alcohol in Islam (Farnia et al., 2024; Michalak & Trocki, 2006). In this study, we observed that 65.3% of boys reported consuming alcohol between once a week and more than seven times a week, contrasting with girls who reported a mere 5% of such consumption. This finding aligns with previous research which indicated that approximately 60% of adolescents had at least one alcoholic drink in the past 30 days (Schiff & Fang, 2014). However, other studies have reported lower levels than what was found in the current study. For instance, in a study involving 2948 Arab Muslim students in Israel, almost 10% of participants reported using alcohol (Eseed & Khoury-Kassabri, 2018), and in a study of 2944 Arab students in Israel, the reported prevalence was 12.1% (Azaiza et al., 2008). Two possible explanations for these differences are (1) over the years, Israeli–Arab adolescents have become increasingly exposed to Israel’s modernization and Westernization, and (2) there may have been variations in the types of populations of Israeli–Arab adolescents in the studies, including differences between those from the northern or southern regions and those residing in the Triangle region.

Additionally, factors such as thought suppression regarding alcohol and self-concealment were identified. Despite reporting alcohol consumption, Israeli–Arab adolescents, influenced by religious and cultural prohibitions (Farnia et al., 2024), often attempt to suppress thoughts related to their drinking. This phenomenon mirrors findings from a study in Iran, where individuals with AUDs showed that negative affect and thought suppression indirectly affected cravings and dependencies through desire thinking (Khosravani et al., 2022). Furthermore, these adolescents frequently conceal their drinking habits from their parents, a behavior linked to problematic alcohol use (Baudat et al., 2022; Chinweuba et al., 2023).

The second factor we found was “CSB, PPU, and mental health,” comprising the measures of CSB, PPU, the prevalence of viewing pornography and sexual behavior, and the measures of well-being and psychological distress.

In this study, 81.6% of boys reported watching pornography and 79.8% engaged in sexual behaviors weekly or more often, while only 48.9% of girls reported watching pornography and 54.4% engaged in sexual behaviors with similar frequency. These findings exceed previous results for Israeli–Arab adolescents, where only 44.4% reported engaging in sexual behaviors and just 31.7% acknowledged using pornography (Jabareen & Zlotnick, 2023). The tension between the Arab community's conservative sexual norms and their exposure to sexual openness creates a profound cultural contrast (Gesser-Edelsburg & Abed Elhadi Arabia, 2018), potentially fostering the development of PPU or CSB alongside adverse mental health outcomes.

The third factor we found was “moral disapproval, thought suppression, and self-concealment of pornography and sexual behavior,” comprising all the measures of moral disapproval and the measures of thought suppression and self-concealment of pornography and sexual behavior. These findings indicate that Arab–Israeli adolescents may face conflicts between their actual engagement in pornography or sexual behaviors and their religious prohibitions against such activities, as observed in this study. This internal conflict often leads to moral incongruence (PPMI; Grubbs & Perry, 2019), where adolescents cope with the resulting negative emotions through moral disapproval, suppressing sexual thoughts, and concealing their behaviors from their parents.

Aside from identifying these three factors, we also found that PPU emerged as the most influential variable in the entire network. Pornography use is prevalent among adolescents (Testa et al., 2023), with frequency of use demonstrating the strongest predictive power of PPU (Bőthe et al., 2024; Chen et al., 2022). In the current study, the frequency of pornography use surpassed that of alcohol consumption and engagement in sexual behaviors. However, relying solely on the frequency of use to determine PPU can introduce biases (Bőthe et al., 2020, 2024). Therefore, we also examined culture-related factors that predict PPU among Israeli–Arab adolescents and found five distinct profiles.

Latent profile analysis uncovered a five-cluster solution that was based on the three detected factors among the study participants: (1) low alcohol use amidst high CSB and moral disapproval, thought suppression, and self-concealment (*n* = 78, 12.5%); (2) low CSB, pornography, and alcohol use (*n* = 111, 17.8%); (3) average CSB, pornography, and alcohol use (*n* = 84, 13.5%); (4) high CSB, pornography, and alcohol use (*n* = 46, 7.4%); and (5) high CSB, pornography, and alcohol use amidst low moral disapproval, thought suppression, and self-concealment levels (n = 303, 48.7%).

The most prevalent profile comprised Israeli–Arab youth with high levels of CSB, pornography, and alcohol use who did not morally disapprove of the acts, and who did not feel the need to self-conceal or suppress the sexual side of their behavior (by contrast, the need to suppress and self-conceal the use of alcohol was high). These findings corroborate earlier research on the prevalence of alcohol (Farnia et al., 2024), pornography, and sexual behavior among adolescents (Jabareen & Zlotnick, 2023). The infusion of Western individualistic values into Arab culture in Israel (Azaiza, 2013) appears to be normalizing these traditionally prohibited behaviors, reducing instances of moral disapproval and the need for concealment from parents. Notably, the reported high levels of CSB may be linked to the ready access to online sexual content (Efrati & Amichai-Hamburger, 2021). While alcohol consumption remains hidden due to religious prohibitions, broader interpretations of “sexual behavior” allow for more openness online, despite restrictions on premarital sex (Ali, 2016; Eseed et al., 2018).

Two other less prevalent profiles comprised adolescents with either below-average or average levels of CSB, pornography, and alcohol use (a total of 31.3%). These profiles pertain to adolescents who have normal engagement with these behaviors, with no signs of a disorder. This result is consistent with prior research suggesting that experimenting with alcohol consumption (Yuen et al., 2020) and engaging in sexual behaviors (Ballester-Arnal et al., 2023) are part of the experience of healthy adolescence. Regarding CSB, previous research has suggested that average levels of CSB among adolescents appear to deviate from the natural course of normative sexual development, as the severity of psychopathology is typically low (Efrati & Dannon, 2019).

The least common profile consisted of Israeli–Arab youth with high levels of CSB, pornography, and alcohol use and a high prevalence of moral disapproval of these acts and the need to self-conceal or suppress the behaviors. Only 7 out of 100 youths showed this profile. This finding supports the notion that cultural factors significantly influence the severity of CSB, pornography use, and alcohol consumption among adolescents. Israeli–Arab adolescents face a pronounced conflict between modern influences that make such behaviors accessible and the strictures of religious and conservative cultural norms (Azaiza, 2013) that prohibit alcohol consumption (Farnia et al., 2024,) pornography viewing, and sexual activities (Ali, 2016; Eljawad et al., 2021; Jabareen & Zlotnick, 2023). This internal conflict often manifests as moral disapproval, with individuals feeling sinful or immoral (Grubbs & Perry, 2019). Research indicates a connection between moral disapproval and behaviors such as CSB (Coleman et al., 2023) and alcohol use (Grynberg et al., 2017). For example, studies among Israeli–Jewish adolescents have shown that moral disapproval, thought suppression related to sexuality (Efrati, 2019; Efrati & Spada, 2024) and self-concealment from parents are significantly linked to increased CSB and other problematic behaviors (Efrati, 2023a).

Finally, another low-frequency profile pertained to adolescents who engaged in CSB and pornography, with the need to self-conceal or suppress the sexual side of their behavior but who spurned alcohol. This profile resembles the previous profile in the context of sexual behaviors, the difference is in the context of alcohol. There are two possible reasons for these findings. The first reason relates to the prohibition in Islam of drinking alcohol (Michalak & Trocki, 2006), a prohibition that these adolescents honored mainly because, for the most part, they self-identified as traditional and/or religious. A second explanation is related to results regarding alcohol that were inconclusive in a previous study (Grubbs, 2022), because the related to self-reported feelings of addiction to alcohol.

Aside from the LPA uncovering a five-cluster solution, we also observed differences related to gender and religiosity. For example, a majority of boys belonged to the “high severity” and “high severity without moral disapproval or thought suppression” groups, whereas the “low use” group had a majority of girls. This finding may align with societal norms that impose greater restrictions on and supervision of girls than boys in the Arab community (Johnston, O’Malley, & Bachman, 2000). Often, societal expectations encourage boys to be more sexually assertive, making it more socially acceptable for them to use sex and alcohol as coping mechanisms (Jabareen & Zlotnick, 2023; Eseed & Khoury-Kassabri, 2018). Additionally, studies have demonstrated earlier and greater engagement in sexual activities among boys than girls (e.g., Meekers & Calvès, 1999).

Similarly, the “high severity without moral disapproval and thought suppression” group had the least religious adolescents in it, whereas the “low alcohol, high CSB” group had the most religious adolescents in it. These findings could be attributed to the strict moral and ethical codes often associated with religious teachings (Haidt, 2012), which may induce a sense of guilt or lack of control when thinking about or engaging in sexual activities (Grubbs & Perry, 2019) and alcohol consumption (Cho & Yang, 2023). Additionally, as stated, the consumption of alcohol is prohibited in Islam (Farnia et al., 2024; Michalak & Trocki, 2006). Regarding CSB, previous studies have already indicated the relationship between religiosity and CSB. For example, a study involving 1500 Israeli–Jewish adolescents found that religiosity was associated with more CSB, particularly in light of the repressed thoughts about sexuality, which were found to be linked with more CSB (Efrati, 2019).

Finally, with respect to age, the “low use” and “low alcohol, high CSB” groups were significantly younger than the “high severity without moral disapproval and thought suppression” group. Previous research has also demonstrated that older adolescents report higher levels of alcohol use and pornography consumption (Carliner et al., 2017; Farré et al., 2020). This finding may be explained by greater exposure to alcohol and sex-related behavior, which are more accessible in the context of social events attended by adolescents, reflecting social acceptance.

### Limitations and Future Research Directions

The current study had a number of limitations, although its main premises were supported. First of all, the study’s correlational nature does not allow for the establishment of causality. Although network analyses and LPA were used, caution must still be exercised when the findings are applied to interventions. Second, self-report measures, which can lead to response biases, especially among this age group, were used. Third, the sample was a moderate-sized convenience sample; larger studies must be conducted in order to validate the findings (e.g., the “high CSB, pornography, and alcohol use” group comprised only 46 participants from the general population). Finally, only Israeli–Arab adolescents participated in the current study. Going forward, to confirm the findings’ replicability and generalizability, other populations (ethnically and culturally diverse) should be investigated.

### Theoretical and Practical Implications

Our findings provide a significant advance in understanding the cultural factors influencing the development of CSB, PPU, and AUDs among Israeli–Arab adolescents. We identified five distinct profiles characterized by mental health issues, moral disapproval, thought suppression, and self-concealment, making a novel contribution to the literature on these subjects. This study is the first to explore the intersections of alcohol, pornography, and sexual behavior in this understudied population, shedding light on how cultural factors such as moral disapproval, thought suppression, and self-concealment impact these behaviors.

Moreover, by examining Israeli–Arab adolescents, we have enriched the existing body of knowledge. Our research highlights the prevalence of drinking alcohol, viewing pornography, and engaging in sexual behaviors, revealing the significant conflict these adolescents face between modern influences and the conservative strictures of their religious and cultural norms. This conflict often leads to moral disapproval, where individuals may feel sinful or immoral, and engage in thought suppression and self-concealment, particularly from parents. Our study enhances understanding of the social dynamics contributing to CSB, PPU, and AUDs, providing valuable insights for researchers, psychotherapists, clinicians, and other mental health professionals. These findings suggest that these behaviors are more closely related to social or cultural factors than to psychopathological ones, offering a foundation for mental health professionals to better support adolescents who experience psychological distress due to their addictive behaviors. Furthermore, identifying culture-related factors in therapeutic settings may improve symptom management and serve as a catalyst for future research into the cultural aspects of CSB, PPU, and AUDs.
